# Fe_3_O_4_@SiO_2_@NiAl-LDH microspheres implication in separation, kinetic and structural properties of phenylalanine dehydrogenase

**DOI:** 10.1016/j.heliyon.2023.e19429

**Published:** 2023-09-03

**Authors:** Mozhgan Amirahmadi, Saman Hosseinkhani, Morteza Hosseini, Paricher Yaghmei, Akbar Heydari

**Affiliations:** a Department of Biochemistry, Faculty of Basic Sciences, Science and Research Branch, Islamic Azad University, Tehran, Iran; b Department of Biochemistry, Faculty of Biological Sciences, Tarbiat Modares University, Tehran 14115-175, Iran; c Department of Life Science Engineering, Faculty of New Sciences & Technologies, University of Tehran, Tehran 1417614418, Iran; d Chemistry Department, Tarbiat Modares University, P.O. Box 14155-4838, Tehran, Iran

**Keywords:** Phenylalanine dehydrogenase, Fe_3_O_4_@SiO_2_@NiAl-LDH microsphere, Kinetic parameters, Protein purification, Activation energy, Thermal stability

## Abstract

Fe_3_O_4_@SiO_2_@NiAl-LDH three-components microsphere contains a Fe3O4@SiO2 magnetic core and a layered double hydroxide with nickel cation provide the binding ability to (His)-tagged-protein and exhibits high performance in protein separation and purification. The morphology and chemistry of the synthesized Fe_3_O_4_@SiO_2_@NiAl-LDH microspheres were characterized by energy-dispersive X-ray spectroscopy (EDX), scanning electron microscopy (SEM), X-ray diffraction (XRD), Fourier transform infrared (FTIR), vibrating sample magnetometer (VSM), Dynamic light scattering (DLS). Purified enzyme was assesed with SDS-PAGE (sodium dodecyl sulfate–polyacrylamide gel electrophoresis and intrinsic fluorescence spectroscopy. In this study, the separation of phenylalanine dehydrogenase (PheDH) by Fe_3_O_4_@SiO_2_@NiAl -LDH was performed and the effect of microsphere was investigated on the kinetic and structural properties of PheDH. After purification, kinetic parameters such as K_m_, V_m__ax_, K_cat_, k_cat/__K__m_, optimum temperature, thermal stability, and and activation energy were evaluated and compared according to the mentioned methods. The interaction between the enzyme and the microsphere displayed a high performance in protein binding capacity. The results also revealed that the kinetic parameters of the enzyme changed in a dose-dependent manner in the presence of a microsphere. Moreover, the results of intrinsic fluorescence and Circular Dichroism (CD) confirmed the structural changes of the protein in the interaction with the microsphere.

## Highlights

• The Protein separation is demonstrated using the high protein binding ability with (Fe_3_O_4_@SiO_2_@NiAl-LDH) magnetic nanoparticles.
• The kinetic and thermochemical parameters of phenylalanine dehydrogenase are compared in presence of Fe_3_O_4_@SiO_2_@NiAl-LDH microsphere
• Fe_3_O_4_@SiO_2_@NiAl-LDH microsphere can be used to separate His-tagged proteins.

## Graphical abstract

In this work, we have exhibited high binding capacity of the phenylalanin dehydrogenase protein with Fe_3_O_4_@SiO_2_@NiAl-LDH microsphere, also we have compared kinetic parameters of phenylalanine dehydrogenase in presence of Fe_3_O_4_@SiO_2_@NiAl-LDH microsphere such as K_cat_, k_cat_/k_m_, k_m_, V_m_, thermal stability, optimum temperature and activation energy by utilizing the spectroscopic method. Also, it has been important to phenylalanine dehydrogenase protein purification via a simple process and using magnetic nanomaterials some advantages such as being readily available, and inexpensive.

**Figure:**
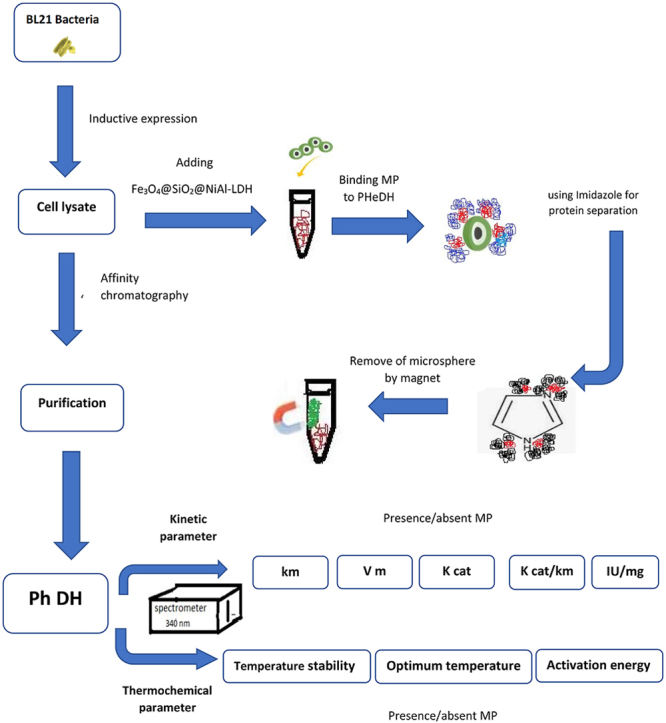

## 1 Introduction

Nowadays, thousands of newborn infants around the world are suffering from an inherited metabolic disorder called Phenylketonuria (PKU). Phenylalanine dehydrogenase with EC (1.4.1.20) is a key enzyme in the diagnosis of phenylketonuria [[1], [2], [3], [4], [5], [6], [7], [8]]. Purification of recombinant proteins is increasingly essential [[9], [10], [11]]. In several life science fields, including proteomics, functional biology, and biopharmaceutics, the expression of target proteins has become significantly important [[12], [13], [14]]. Due to their low immunogenicity, small size, and widespread application (His) -tagged proteins, which include six consecutive histidines, are considered for production [[15], [16], [17]]. The His-tag can coordinate with immobilized transition metal ions (Cu^2+^, Ni^2+^, Zn^2+^, and Co^2+^) using metal-chelating agents such as iminodiacetate (IDA) [18,19], nitrilotriacetate (NTA) [20,21], and tris(carboxymethyl)ethylenediamine (TED) [22,23]. Traditional methods use affinity chromatography to purify (His)-tagged proteins using columns such as Sepharose, which nickel ions are immobilized on it [[24], [25], [26]]. The use of this technique has disadvantages such as long separation time, high-pressure drop, and low diffusion of solutes in the particles [27,28]. Histidine is one of the most widely used affinity tags that could be readily incorporated into desired proteins via expression of the target gene in microorganism cells using commercial expression vectors [29]. Conventional His-tagged protein separation normally takes place in columns employing nickel nitrilotriacetic acid (Ni-NTA) beads; however, this method has significant drawbacks, such as the necessity for pretreatment to remove cell waste and colloidal impurities, lengthy operating time, and protein solubility. Affinity particle adsorbents are a potential new separation nano-tool that has just recently come into being for the separation of proteins [[30], [31], [32]]. Magnetic nanoparticles have significant advantages over typical micrometer-sized resins or beads for these bio separation applications due to their small size and high surface area, including strong dispersibility, quick and efficient binding of biomolecules, and regeneration [33]^.^

In recent years a few researchers have investigated the possibility of magnetic nanoparticles application for the purification of proteins [34,35]^.^ Furthermore, magnetic separation offers exceptional opportunities for the straightforward, efficient, and quick concentration of purified specific proteins, cells, and other biomolecules without subjecting them to potentially harmful chemical and physical treatment. Magnetic separation is a quick and highly efficient method [36,37]. The creation of MNPs with a core-shell structure is a beneficial technique. In addition to significantly enhancing the stability of the magnetic core, the core-shell structure provides a useful framework for immobilizing a range of functional groups to satisfy the numerous demands of biological separation applications [38,39]. Targeted molecules can be quickly captured when magnetic nanoparticle surfaces are functionalized to provide surfaces that are particular to a certain substrate. Consequently, the separation and purification of biomolecules including proteins, nucleic acids, and enzymes could be performed quickly and easily using magnetic separation, which does not require pretreatment procedures (such as centrifugation or filtering), also due to its high binding capacity to proteins, the mentioned magnetic microsphere can be used in design and construction of biosensors for the diagnosis of genetic diseases. In addition, magnetic particles can be used to design and build non-invasive tools to detect viral diseases such as Covid-19 due to their high protein binding capacity [[40], [41], [42]]. Separation systems based on magnetic nanomaterials have been used to reduce the disadvantages of columns such as Fe_3_O_4_/SiO_2_ microspheres [43,44], nitrilotriacetic acid [NTA]and or use of Au–Ni–Au triblock nanorods [45,46]. Also, other systems have been developed such as Ni/Ni core-shell nanoparticles for the magnetic separation of histidine-tagged proteins [47]. However, there are still drawbacks, such as poor recyclability and low magnetic moments for the use of these materials in the separation of proteins [48]. Therefore magnetic separation of proteins is a required system with appropriate magnetic properties, high capacity, and recyclability [49]. Double hydroxides are a group of inorganic compounds with a lamellar structure and are known as hydrotalcite-like compounds [50].

In recent years, these materials have shown different applications due to their special structure such as adsorbents [[51], [52], [53]], catalysts [[54], [55], [56]], as a precursor in metal oxides [[57], [58], [59], [60]], separation [61,62], biology, medicine [[63], [64], [65], [66]]. There are different methods for synthesizing LDHs such as the co-precipitation method [[67], [68], [69]], and other methods such as the sol-gel method [[70], [71], [72]], hydrothermal synthesis method [73], and urea hydrolysis method [[74], [75], [76]]. The co-precipitation method has several advantages, such as high purity and crystallinity. Factors affecting well-crystallized hydrotalcite or how the shape of amorphous materials are solution concentration, pH, nature of the solution, cation concentration, temperature, aging time, and cation molar ratios [77]. There are still challenges in using this material although several structures have been developed so far, such as 3D microporous LDHs [[78], [79], [80], [81]], functional ultrathin films [82,83], and hollow spheres or core-shell structures [[84], [85], [86]]. The Fe_3_O_4_@SiO_2_@NiAl -LDH microspheres demonstrate super binding and selectivity to His-tagged proteins in intricate biosystems. These MPs could be utilized directly to extract His-tagged proteins from the mixture of lysed cells, similar to other structures [[87], [88], [89]]. In this study, efficiency of protein separation by Fe_3_O_4_@ SiO_2_@ Ni Al-LDH microspheres was evaluated by interacting with His-tagged proteins. In addition, this report considered the changes in kinetic parameters and the activation energy of the enzyme that was calculated and compared in the presence of the aforementioned magnetic microspheres. Finally, the optimum temperature and thermal stability of the native enzyme are compared in the presence of the Fe_3_O_4_@SiO_2_@NiAl -LDH microspheres.

## 2 Result and discussion

### 2.1 Characterization of the Fe_3_O4@SiO_2_@ Ni Al-LDH microsphere

#### 2.1.1 SEM and EDX analysis

**Fig. 1:**
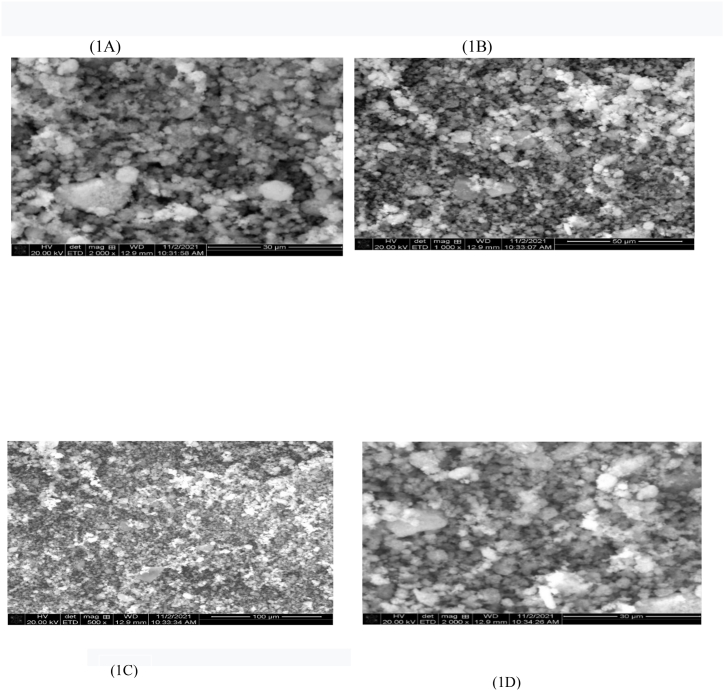
SEM image demonstrates the size of microspheres at different scales (1A–1D).

**Fig. 2:**
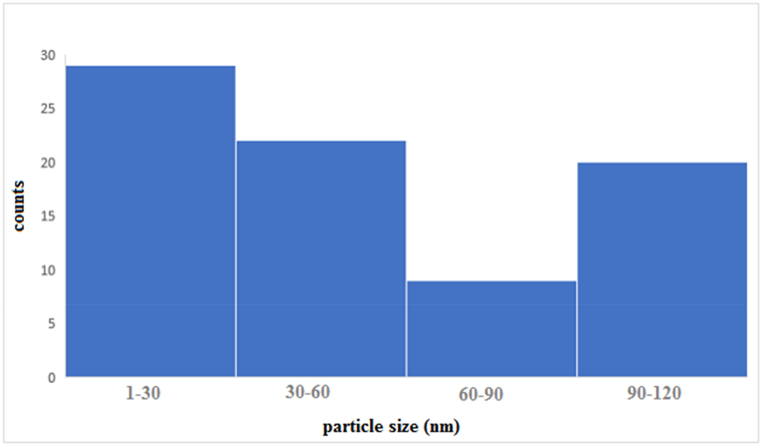
Particle size distribution of synthesized microspheres..

Fe_3_O_4_@SiO_2_@ Ni–Al LDH was synthesized based on the mentioned method in previous studies via a solvothermal method. The appearance of the microsphere was investigated by scanning electron microscopy (SEM) (Fig. 1) at different scales. The maximum and minimum sizes were 93.8 and 16 nm, respectively (Fig. 2). SEM images show uniform flowerlike shapes of the microsphere. It is worth mentioning, without the presence of AlOOH coating cannot be obtained. Fe_3_O_4_@SiO_2_@ Ni Al -LDH core-shell and ALOOH plays an important role in providing Al for LDH nanocrystal growth.

Particle size distributions were determined and the average particle size was plotted against the number of particles. The particle size diagram was evaluated by measuring the number of 120 particles, the graph shows the maximum and minimum particle size distribution 29 and 9 nm, respectively (Fig. 2).

#### 2.1.2 X-ray spectroscopy (EDX)

**Fig. 3:**
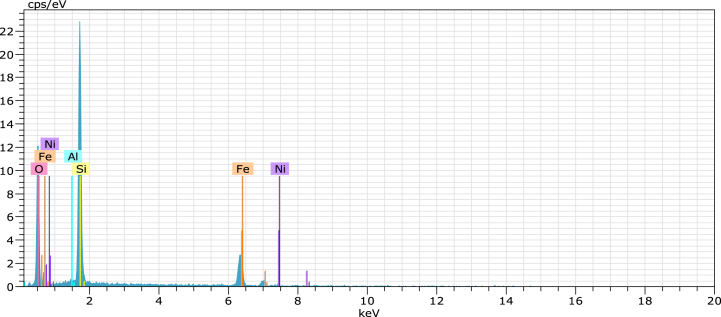
The EDX spectrum of the microsphere (MP).

EDX displayed the modification of the Fe_3_O_4_@SiO_2_ surface by the Ni Al-LDH also, The EDX map analysis showed the presence of Al, O, Fe, Si, and other elemental contents (Fig. 3 and Table 1).

**Table 1:** Elemental content analysis of microsphere.

El	AN	Series	unn. C [wt.%]	norm. C [wt.%]	Atom. C [at.%]	Error (1 Sigma) [wt.%]
O	8	K-series	38.81	51.29	70.43	6.42
Si	14	K-series	19.74	26.08	20.40	0.92
Fe	26	K-series	16.65	22.00	8.65	0.65
Al	13	K-series	0.47	0.62	0.51	0.08
Ni	28	K-series	0.00	0.00	0.00	0.00
		Total:	75.67	100.00	100.00	

#### 2.1.3 XRD analysis

**Fig. 4:**
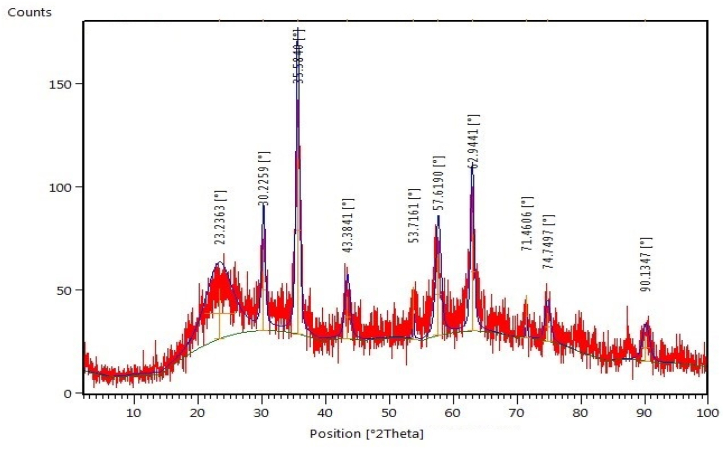
XRD pattern for Fe_3_O_4_@SiO_2_@ Ni Al-LDH.

The XRD pattern was demonstrated in (Fig. 4 and Table 2). The average crystallite size was calculated by the Scherrer equation for the 3 Main peaks of Fe_3_O_4_@SiO_2_@ Ni Al-LDH which was 8.8 nm in size. The peaks of diffraction in a curve can be indicated as a face-centered cubic (fca) for the Fe_3_O_4_ phase also the pattern of diffraction shows a characteristic of amorphous for SiO_2_ [11], as well as, Fe_3_O_4_ Cubic structure reference (JCPDS card no. 96-900-5839). The XRD patterns of Fe_3_O_4_@SiO_2_@AlOOH synthesis showed the diffraction angle of 30.22°, 35.58°, 43.38°, 57.61°62.94°and 74.74°. The XRD pattern of Fe_3_O_4_@ SiO_2_@ Ni Al-LDH microsphere is similar to Fe_3_O_4_@SiO_2_ which indicates the crystal structure of boehmite but the XRD pattern after situ growth and formation of microsphere showed high crystallinity of the product.

**Table 2:** Shows the result of the XRD in terms of Angstrom unit.

No.	B obs. [°2Th]	B std. [°2Th]	Peak pos. [°2Th]	B struct. [°2Th]	Crystallite size [Å]
1	0.880	0.088	35.620	0.792	105
2	1.260	0.126	62.870	1.134	82
3	1.300	0.130	57.360	1.170	77

#### 2.1.4 FT-IR spectroscopy

**Fig. 5:**
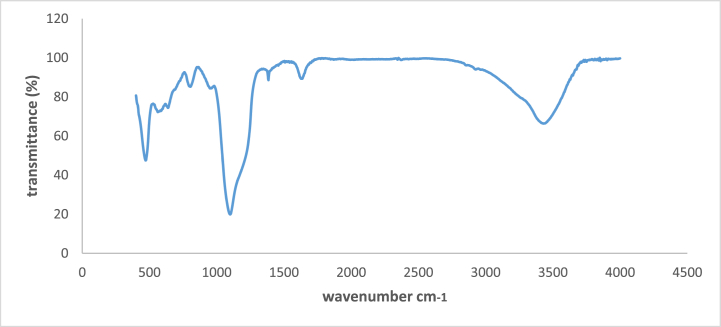
FT-IR spectra of Fe_3_O_4_@SiO_2_@ Ni Al LDH.

The construction of the Fe_3_O_4_@SiO_2_@NiAl-LDH microsphere was examined using FT-IR spectroscopy, which confirmed the presence of anticipated functional groups. Fig. 5 shows the FT-IR spectrum for Fe_3_O_4_@SiO_2_@ Ni Al LDH microspheres. Peaks 471 and 591 cm^−1^ both expressed the existence of Fe–O and the production of Fe_3_O_4_ was indicated by peaks at 1629 cm^−1^. In addition, the result shows that Fe_3_O_4_@SiO_2_@Ni Al-LDH microspheres could be dispersed in water by sonication or vigorous shaking and redispersion in the presence of an external magnetite field. The microspheres possess high magnetic susceptibility which can play an important role in practical manipulation [90,91]^.^

#### 2.1.5 VSM analysis

**Fig. 6:**
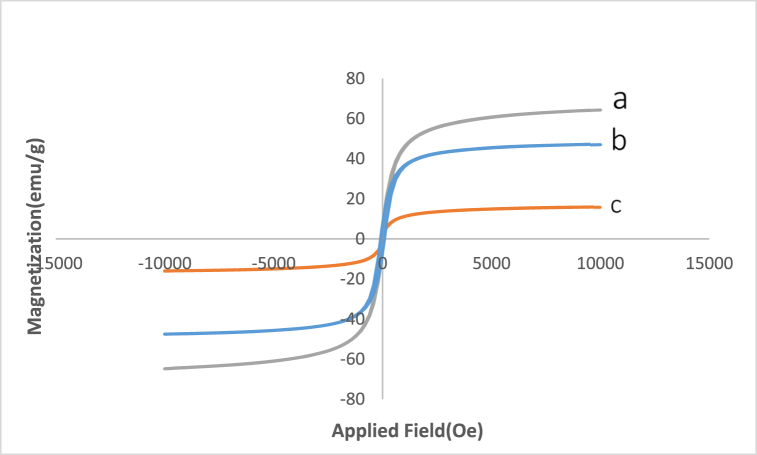
VSM analysis of (a) Fe_3_O_4__;_ (b) Fe_3_O_4_@SiO_2_ and (c) Fe_3_O_4_@SiO_2_@ Ni Al-LDH.

VSM analysis was evaluated to determine the magnetic behavior of the Fe_3_O_4_@SiO_2_@ Ni Al LDH hybrid. As shown in Fig. 6, magnetic measurements at ambient temperature from −10,000 to +10,000 showed that the saturation magnetization (Ms) value of the Fe_3_O_4_@SiO_2_@ Ni Al LDH compared to that of Fe_3_O_4_@SiO_2_ was lowered to 32 emu g^−1^, which was attributed to the addition of Ni Al LDH to reducing the magnetization of Fe_3_O_4_@SiO_2_. Also, the magnetization (Ms) of Fe_3_O_4_ (64 emu g^−1^) and Fe_3_O_4_@SiO_2_NPs (47 emu g^−1^) was higher than the Fe_3_O_4_@SiO_2_@ Ni Al LDH microsphere (15. emu g^−1^); which was confirmed the superparamagnetic nature of all three examined samples. As well as, even when Ms was reduced, the composite microsphere still showed strong magnetic activity. It could be conveniently collected from the reaction mixture using an external magnet.

#### 2.1.6 Intrinsic fluorescence spectroscopy

**Fig. 7:**
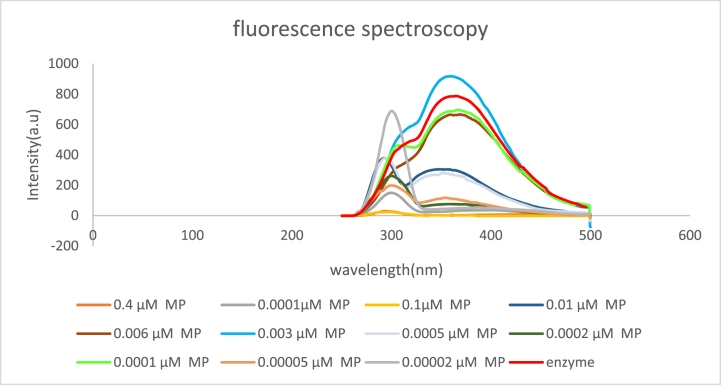
Enzyme fluorescence intensity in the presence of different concentrations of microsphere.

Fluorescence spectroscopy has been performed to investigate changes in the intrinsic fluorescence of the enzyme in the presence of microspheres. Fig. 7 shows the intrinsic fluorescence changes of the PheDH enzyme in the presence of a different concentration of microspheres. It is worth mentioning that the fluorescence intensity of the enzyme was changed in the presence of microspheres. It seems that microspheres can cause a change in the tertiary structure of the protein. Previous studies have revealed that the change in the environment of Trp120, which is located between regulatory and catalytic domains, can cause a change in the intensity of fluorescence spectroscopy [92]. Also, a similar phenomenon was observed in published work on the assessment of the tertiary structure changes by analyzing the intrinsic fluorescence of mutant and native phenylalanine dehydrogenase obtained from *Bacillus badius*, have shown that change in the emission spectrum resulting from tyrosine can be a response to local changes around the indole ring of tyrosine and the mutant proteins show less fluorescence emission at 340 which was attributed to conformational changes of protein structure [93]. Also, the study has shown that the interaction of the CdTe quantum dot with PHDH enzyme in a dose-dependent concentration can change the intrinsic fluorescence emission of the enzyme [94].

#### 2.1.7 The zeta potential and dynamic light scattering (DLS)

**Fig. 8:**
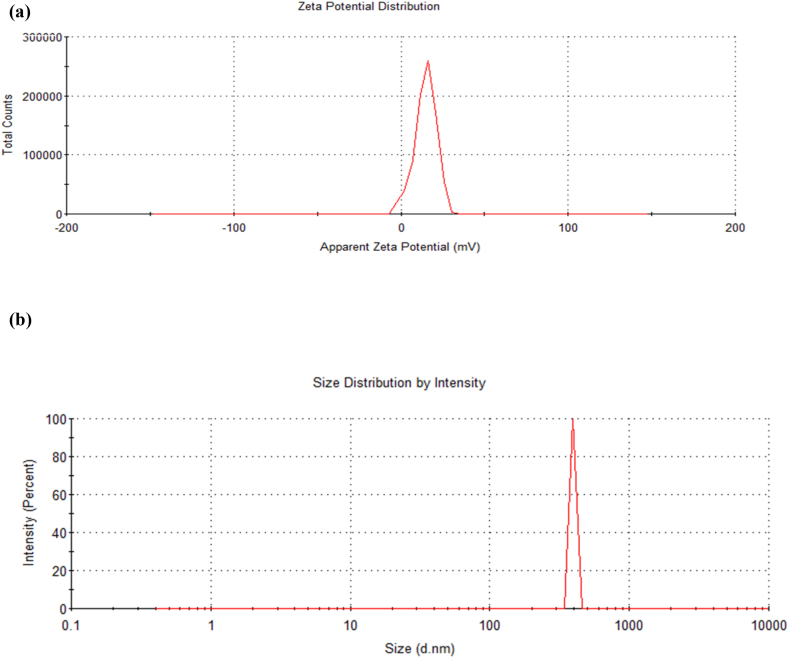
(a)The zeta potential for Fe_3_O_4_@SiO_2_@ Ni Al-LDH microsphere. (b) Average size of Fe_3_O_4_@ SiO_2_@ Ni Al LDH microsphere.

The zeta potential was carried out to determine the surface charge. The zeta potential distribution spectra were shown for Fe_3_O_4_@SiO_2_@ Ni Al LDH (Fig. 8a) which was determined 14.6 mv (Table S1), also the average size of microspheres was determined 396.1 nm (Fig. 8b and Table S2). The studies have shown that the zeta potential values were determined for Fe_3_O_4_@SiO_2_@ Ni Al-LDH microsphere 26 mv and the other microspheres that contained Co, Zn, and Mg metals determined 36.9, 24.2, 17 mv respectively. It seems that there is no correlation between surface potential and adsorption capacity among the mentioned microsphere [11].

### 2.2 Separation of phenylalanine dehydrogenase by the Fe_3_O_4_@SiO_2_@Ni Al-LDH microsphere

#### 2.2.1 Circular dichroism (CD)

**Fig. 9:**
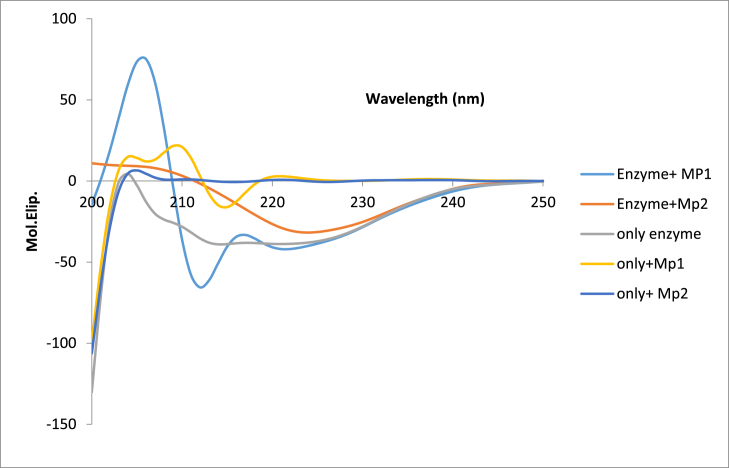
Circular Dichroism (CD) spectra of enzyme in the absence and presence of 0.7 mg ml^−1^ and 1 mg ml^−1^ of microsphere (MP1 = 1 mg ml^−1^ and MP2 = 0.7 mg ml^−1^).

The interaction of magnetic nanoparticles with proteins could lead to a change in protein conformation. The purpose of CD in this research was to investigate and compare the changes in the secondary structure (alpha helix and beta sheet contents) of protein in the presence of microspheres. The circular dichroism (CD) demonstrated changes in the secondary structure of the enzyme (α helix and β strands) in the presence of two different concentrations of Fe_3_O_4_@SiO_2_@ Ni Al-LDH microsphere (Fig. 9) Spectra analysis of result showed that the percentage of α helix decreased but β strand increased in the presence of microsphere (Table 3). Therefore, the secondary structure of the protein can be changed in the presence of the Fe_3_O_4_@SiO_2_@ Ni Al-LDH microsphere. A similar phenomenon was shown in published research on CD analysis resulting from the interaction of iron oxide nanoparticles (SPIONs) with iron saturated human transferrin protein that has revealed irreversible changes in the protein structure [95]. Changes in protein conformation can modify their function and help in recognizing the molecular mechanisms of injury and the pathogenesis of the disease [[96], [97], [98]].

**Table 3:** Analysis of secondary structure of the enzyme in the presence of different concentration of Fe_3_O_4_@SiO_2_@ Ni Al -LDH microspheres (MP).

Enzyme	α helix (72.9%)	β strand (3.07%)
Enzyme + MP1	6.16%	37.07%
Enzyme + MP2	32.98%	16.06%

#### 2.2.2 Protein separation

Efficiency in protein separation of Fe_3_O_4_@ SiO_2_@ Ni Al-LDH microspheres was evaluated by their reaction with His-tagged PDH proteins. Fe_3_O_4_@ SiO_2_@ Ni Al-LDH microspheres was incubated with His-tagged proteins under certain conditions and finally separated from the solution by a magnet. Imidazole solution with a certain concentration was used to release the protein. Finally, SDS-PAGE was used to confirm PheDH separation and protein concentration determined using Bradford method. In Fig. 10A; the steps of protein separation by use of Fe_3_O_4_@ SiO_2_@ Ni Al-LDH microsphere is presented, schematically. Table 4 shows the amount used for protein separation which indicates an excellent interaction between the microsphere and PheDH enzyme.

**Fig. 10A:**
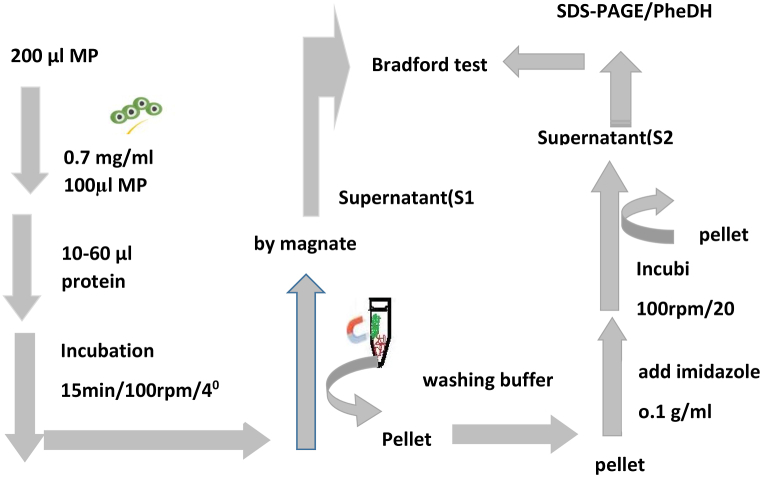
Schematic representation of the reversible separation of phenylalanine dehydrogenase by Fe_3_O_4_@ SiO_2_@ Ni Al-LDH microsphere.

**Table 4:** Volumes and concentrations used in determining the binding capacity.

Enzyme, C (mg/ml)	MP, C (mg/ml)	Enzyme, V (μl)	MP, V (μl)
0.78	0.7	10	100
0.78	0.7	20	100
0.78	0.7	30	100
0.78	0.7	40	100
0.78	0.7	50	100

#### 2.2.3 Determining the binding capacity

Fig. 10B and Table 4 show respectively the method and values used in determining the binding capacity of the enzyme with the microsphere. As shown in Fig. 10C, the interaction between the enzyme and Fe_3_O_4_@SiO_2_@ Ni Al-LDH microsphere under certain conditions exhibited excellent performance in the separation of protein with a binding capacity of 241 μg/mg.

**Fig. 10B:**
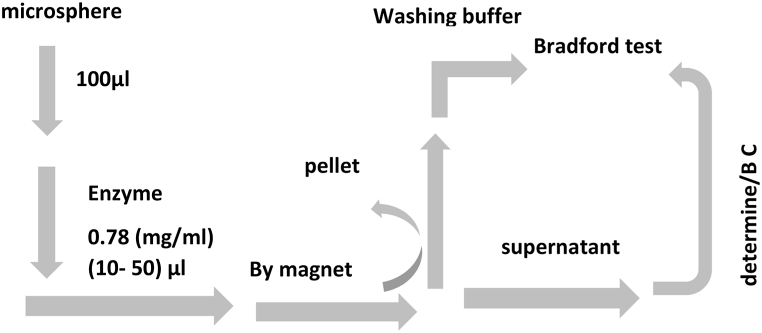
The scheme shows the procedure for determining the binding capacity of the enzyme.

**Fig. 10C:**
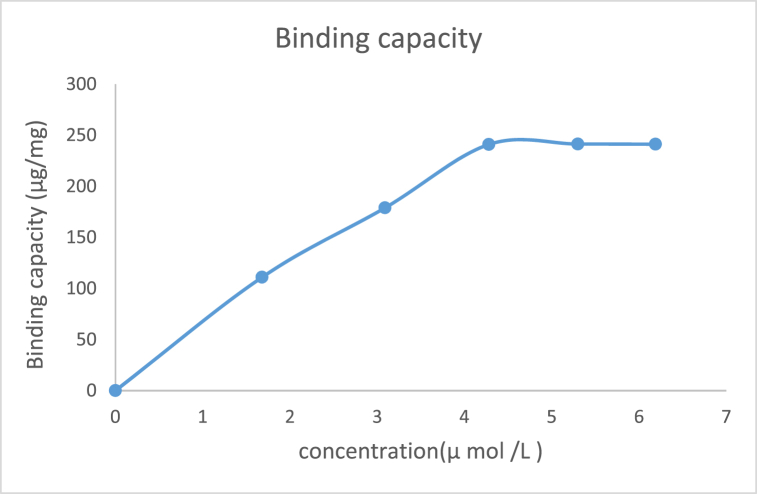
Enzyme binding capacity of microspheres at different enzyme concentration.

Magnetic nanoparticles, containing transition metal ions, possess the ability to bind recombinant proteins that attach to the C- or N-terminal histidine and magnetically separate (His-tagged) proteins from lysed cells. Binding capacity is a characteristic that shows the maximal amount of adsorbate bound onto a surface through chemical or physical reaction [99]. Binding capacity has reported 239 μg/mg in the separation of (His)-tagged green fluorescent protein by Fe_3_O_4_@ SiO_2_@ Ni Al-LDH microsphere [11]. Evidence has shown that increasing the adsorbent surface and modifying it can increase the binding capacity. A separation study of green fluorescent protein (GFP) by modified Fe_3_O_4_ magnetic nanoparticle has revealed that 0.032 mmol of GFP could be bound to each gram of modified Fe_3_O_4_ while the surface area of the nanoparticles was 97 m^2^/g. Investigations have shown that changing the pH can change the adsorption capacity of the enzyme on magnetic nanoparticles, so that the maximum adsorption occurred at the isoelectric pH (P*I*) of the enzyme [100,101]. Among other factors that can change the adsorption rate and binding capacity of nano magnetic particles is the incubation time factor, so by changing this time, capacity binding can be changed [102].

#### 2.2.4 The kinetic parameters

The kinetic parameters of the enzyme were determined and compared in the presence of Fe_3_O_4_@ SiO_2_@ Ni Al-LDH microspheres. Three different and consecutive concentrations of microsphere (0.006, 0.013, 0.026) μM were used to compare K_m_, V_max_, k_cat_/K_m_ and enzyme activity in the presence of microsphere. The results showed that with a gradual increase in microsphere concentration Km and Vmax decreased while the enzyme efficiency (kcat/Km) increased. Also, the results showed that high enzyme activity can be obtained from the effect of increasing the microsphere concentration (Table 5, Fig. S7. Fig. S8, Fig. S9, Fig. S10). It seems that the presence of the nano magnetic particles can modulate the catalytic activity of the attached enzyme and change enzyme activity in reaction [103,104]. In addition, magnetic nanoparticles can also affect the stability of the enzyme. The interaction and immobilization of yeast alcohol dehydrogenase on superparamagnetic Fe_3_O_4_ have shown 10 fold more stability and 2.7 fold increased enzyme activity compared to the free enzyme [105]. Examining the interaction between nanoparticles and phenylalanine dehydrogenase from *Bacillus badius* has shown an inhibitory role on the kinetic parameters of the enzyme with increasing concentration of nanoparticle [106]. Evidence has confirmed that magnetic nanoparticles can change the enzyme's activity and stability by changing the conformation of the enzyme.

**Table 5:** Kinetic parameters of enzyme in the absence and in the presence of Fe_3_O_4_@ SiO_2_@ Ni Al-LDH.

	K_m_ (mM)	V_m_ (mmol.min^−1^)	K _cat_ (S^−1^)	K _cat_/Km (S^−1^.m M^−1^)	IU	IU/mg
PheDH	0.10	2.28	126	1260	3.84	760
PheDH + 0.006 μM MP	0.72	2.72	151	209	2.72	540
PheDH + 0.013 μM MP	0.56	2.14	119	212	2.76	552
PheDH + 0.026 μM MP	0.47	2.09	116	246	3.04	608

#### 2.2.5 Optimum temperature

The optimum temperature of the enzyme was determined in the range of (10–80) °C, in the presence and absence of microspheres. The result showed that the optimum temperature of the enzyme did not change significantly in the presence of the microsphere (Fig. S11). Also, results demonstrate the relative activity of the enzyme in comparison with the presence of Fe_3_O_4_@SiO_2_@ NiAl-LDH microsphere (Table 6). Magnetic nanoparticles can change the optimum temperature of the enzyme in the reaction conditions [107,108]. It should be noted, in another study, the optimal temperature of firefly luciferase was increased to 30 °C by functionalized magnetic nanoparticles [109].

**Table 6:** Relative activity of the enzyme in the absence and presence of microspheres at different temperatures..

Enzyme		
Temperature (°C)	ΔA	Relative activity%
10	0.07	29.4
20	0.2	87.7
30	0.22	97.4
40	0.23	100
50	0.22	96.0
60	0.18	77.6
70	0.11	49.1
80	0.08	35.5
Enzyme and MP		
Temperature ( °C)	ΔA	Relative activity%
10	0.08	41.1
20	0.13	63.9
30	0.17	87.8
40	0.2	100
50	0.19	97.9
60	0.17	85.3
70	0.15	79.2
80	0.05	24.4

#### 2.2.6 Comparison of thermal stability

As we know, temperature can play a key role in maintaining enzyme stability. Results show a change in thermal stability for the enzyme in the presence of Fe_3_O_4_@ SiO_2_@ Ni Al-LDH microsphere at 45 °C and 50 °C, respectively. The temperature stability of the enzyme in the presence of Fe_3_O_4_@ SiO_2_@ Ni Al-LDH microsphere was determined by measuring the relative activity

(Table 7 and Table 8). Results show that the enzyme maintains 48% of activity at 45 °C for 50 min, activity of the enzyme exposed to two different temperatures (45 °C and 50 °C) in an hour but in the presence of a microsphere can maintain more than 50% of activity for 70 min (Fig. S11). Moreover, results demonstrate that the enzyme maintains less than 44% of activity at 50 °C for 50 min whereas in the presence of microspheres can maintain more than 40% of activity at 50 °C for 70 min (Table 8, Fig. S12). Results highlight the role of microspheres in the thermal stability of the enzyme. Magnetite nanoparticles can increase the thermal stability of the enzyme. Magnetic gold mesoporous silica nanoparticle core shells (mAu@PSNs) have been able to increase the activity and thermal stability of cellulase so that enzyme separation could be easily carried out by a magnet despite covalent bonds [110]. A cellulase treated with Fe_3_O_4_ nanoparticles has revealed extended thermal stability in the range of 50–60 °C up to 12 h [111]. It seems that regardless of the type of connection, nano magnetite particles can cause significant changes in the thermal stability of the enzyme.

**Table 7:** Relative activity for enzyme in the ansence and presence of 0.026 mM of microsphere at 45 °C.

Enzyme + MP		
Time (min)	ΔA	Relative activity%
10	0.135	100
20	0.31	98.4
30	0.303	96.2
40	0.293	93.0
50	0.274	86.9
60	0.2	67.6
70	0.16	50.1
80	0.04	11.7
Enzyme		
Time (min)	ΔA	Relative activity%
5	0.2	100
10	0.19	98.0
20	0.19	94.0
30	0.18	88.0
40	0.16	82.1
45	0.16	79.6
50	0.1	48.2
55	0.04	19.4
60	0.01	5.8

**Table 8:** Relative activity for the enzyme in the absence and the presence of 0.026 mM of microsphere at 50 °C.

Enzyme + MP		
Time (min)	ΔA	Relative activity%
10	0.28	100
20	0.275	97.2
30	0.27	94.3
40	0.26	92.9
50	0.24	83.4
60	0.16	55.8
70	0.12	42.4
80	0.01	4.9
Enzyme		
Time (min)	ΔA	Relative activity%
5	0.12	100
10	0.12	94.4
20	0.11	91.2
30	0.11	88.8
40	0.1	80
45	0.09	72
50	0.05	44.8
55	0.01	7.2
60	0.007	5.6

#### 2.2.7 Activation energy

**Fig. 11:**
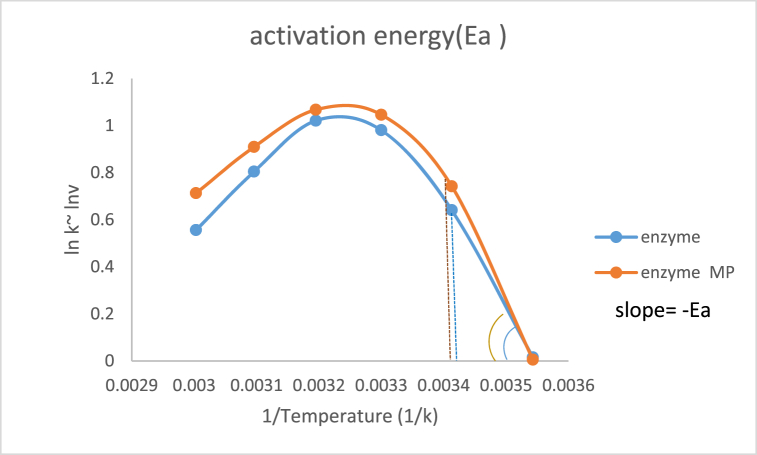
Activation energy of native enzyme and in the absence and presence of microsphere.

In this work, we calculated the enzyme activation energy in the presence and absence of the microsphere using the Arrhenius equation. Fig. 11 shows the comparison of the activation energy from the slope of the Arrhenius plot for native enzyme and enzyme in the presence of microspheres. The enzyme activation energy was calculated at 40.2 kJ/mol but in the presence of microsphere increased to 47 kJ/mol. Modifying agents or treatment with nanoparticles can change the activation free energy of the enzyme and improve the thermostability. Motivated B-cyclodextrin derivatives as modifying agents for PheDH from *Bacillus badius* have revealed that the activation free energy increased by 16 kJ/mol after glycosylation [112]. The activation energy for PheDH from the *Methylotroph nocardia* sp.239 was calculated from an Arrhenius plot and found to be 44 kJ/mol [113]. Changes in Phenylalanine dehydrogenase activation energy upon immobilization has reported for the *sporosarcina ureae* enzyme in which free and immobilized enzyme activation energy was 44.48 and 48.04 kJ/mol respectively [114].

## 3 Experimental details

### 3.1 General

#### 3.1.1 Sample characterization of microsphere

All chemicals, reagents, and solvents used in experiments were purchased from the Sigma Aldrich and Merck companies. Also, SEM was used to investigate microsphere morphology (BRUKER X Flash 6I10I) with a voltage of 20 kv that was combined with x-ray spectroscopy (EDX). Shimadzu IR-470 spectrometer in transmission mode with KBr pellets of the sample was used to record FTIR Spectra.

The XRD analysis was carried out by Rigaku-X-ray diffractometer (model: ULTIMA IV, Rigaku, Japan) with Cu Kα radiation at a voltage of 40 kV, Ampere 40 mA, step size 0.04°, and a scan rate of 4° per minute. The zeta potential analysis was carried out using a Malvern Instrument Zetasizer. Structural changes were investigated by Circular dichroism spectra on a JASCO – 715 spectropolarimeter. Intrinsic fluorescence emission spectroscopy was measured by PerkinElmer LS-55 fluorescence spectrometer. The magnetic properties of the prepared samples were achieved at room temperature by VSM analysis, accomplished via LBKFB model-magnetic Kashan Kavir.

### 3.2 Expression, purification, kinetic and thermochemical studies

#### 3.2.1 Expression and purification of PheDH

*B. badius* PheDH encoding plasmid (pET28a Plasmids) were transformed to *E. coli* BL21 as reported in a previous study. The first 10 μl of LB culture medium was incubated overnight with 50 μg/l kanamycin at 37 °C and 180 rpm. Then 1 ml of seeding culture was added to 250 ml of 2xyt culture medium with kanamycin and incubated at 37 °C and 180 rpm for 4 h. When OD_600_ reached 0.6–0.8, lactose (8.0 mM) was added to 250 ml of 2xyt, and the mixture was incubated at 18 °C and 180 rpm for 16 h. After that, the cell pellet was collected and separated from the supernatant by centrifugation at 15000 for 10 min at 4 °C. The suspended pellet cells were dissolved in lysis buffer (pH 7.4), then the suspension was sonicated on ice. The procedure for performing sonication was cycle of 20 s of sonication and 40 s of rest per minute, which was repeated 13 times. Supernatant collected by centrifugation of 14,000*g* for 20 min at 4 °C. At last step, supernatant was purified by Ni-NTA-Sepharose affinity chromatography column with 250 mM imidazole as elution buffer. Coomassie blue and serum albumin was used as a standard Bradford protocol for determining protein concentration.

#### 3.2.2 Enzyme assay

The activity of PheDH and kinetic parameters of the enzyme were determined at 25 °C, 100 mM glycine-KOH buffer (pH 10.5). Oxidative deamination was assayed by measuring the reduction of NAD+ at 340 nm. Kinetic parameters of the enzyme were obtained using spectrophotometric assay (Biochrome Serial number 114147 8v Dc UV–Vis spectroscopy) at 25 °C with fixing the concentration of NAD^+^ at 2.5 mM while l-phenylalanine concentration was varied, also reaction mixture contained appropriate amounts of enzymes in a total volume of 250 μl.

#### 3.2.3 The optimum temperature

The optimum temperature of the enzyme is obtained with enzyme assay in a range of temperatures between 10 and 80 °C. The mixture contained l-phenylalanine amino acid, NAD^+^, glycine-KCl (pH 10.5) then the mixture was heated in a water bath at mentioned temperature for 5 min. Then enzyme at an appropriate volume was added to the mixture in a total volume of 250 μl and absorbance was measured using spectrophotomete at 340 nm. The optimum temperature of enzyme activity in the presence of a microsphere was determined by the above method. At first, microspheres were prepared at 0.02 μM concentration, then an equal molar of the microsphere was added to each of the primary mixtures (l-phenylalanine, amino acid, NAD^+^, glycine-KCl buffer pH 10.5) in the total volume of 250 μl. Absorbance measurements were obtained using Biochrome Serial number 114147 8v Dc UV–Vis spectroscopy.

#### 3.2.4 Thermal stability

Thermal stability was determined using the spectroscopic method for PheDH in the presence and absence of microspheres at 340 nm. A certain amount of the enzyme was heated in a water bath at the desired temperature and for a certain period, then the enzyme was cooled on ice for a few minutes, and absorption of the mixture was measured. It should be emphasized that the assay cocktail contains glycine-KCl buffer (pH 10.5), l-phenylalanine, NAD^+,^ and a suitable amount of the enzyme in a total volume of 250 μL. The thermal stability of the enzyme was measured as described above in the presence of microspheres. An appropriate volume of microspheres was added to the reaction mixture and the absorption of the reaction mixture was measured in total volume 250 μl by using Biochrome Serial number 114147 8v Dc UV–Vis spectroscopy at 340 nm.

#### 3.2.5 Activation energy

Activation energy calculated from Arrhenius plot slope in the temperature ranges of 10–60°. The reaction mixture contained l-phenylalanine amino acid, NAD^+^, glycine-KCl (pH 10.5) buffer, and a proper amount of enzyme in the total volume of 250 μl. Absorbance was measured for each of the mixtures in the mentioned temperatures at 340 nm by fixing the value of NAD^+^, keeping the phenylalanine concentration variable. Each sample was evaluated by using Biochrome Serial number 114147 8v Dc UV–Vis spectroscopy. Maximum velocity (Vmax) obtained from line weaver-burk plot thenslope of lnV_max_ vs 1/T from the Arrhenius plot and Activation energy was obtained.

#### 3.2.6 Fluorescence spectroscopy

Intrinsic fluorescence emission spectroscopy was measured in the presence of different concentrations of Fe_3_O_4_@SiO_2_@NiAl -LDH at room temperature. The excitation wavelength of the enzyme was 295 nm and the range of the emission spectrum was 250–500 nm. The width of the exciting slits was 5 and 10 and the protein concentration for each assay was 0.7 mg ml^−1^ in the total aliquot of 60 μl. Intrinsic Fluorescence emission spectroscopy was measured by PerkinElmer LS-55 fluorescence spectrometer.

#### 3.2.7 Synthesis of Fe_3_O_4_@ SiO_2_@ Ni Al-LDH

All procedures of Fe_3_O_4_@ SiO_2_@ Ni Al LDH microspheres synthesis, including preparation of uniform magnetite Fe_3_O_4_, preparation of Fe_3_O_4_ structures by sol-gel method, deposition of ALOOH on the surface of Fe_3_O_4_@ SiO_2_ via layer by layer method, LDH growth on the surface of Fe_3_O_4_@SiO_2_@AlOOH with a solution containing M^2+^ ions carried out according to the previous methods.

#### 3.2.8 Phenylalanine dehydrogenase binding and separation

##### 3.2.8.1 Binding capacity

Interaction of Fe_3_O_4_@ SiO_2_@ Ni Al LDH microsphere with Phenylalanine dehydrogenase and determination of enzyme binding capacity. Fe_3_O_4_@ SiO_2_@ Ni Al LDH microspheres (0.7 mg) were added into 1 ml distillation water then the enzyme with 0.78 mg/ml concentration was added into a fixed volume of the microsphere. The reaction mixture contained PheDH/glycine-KCl, Fe_3_O_4_@ SiO_2_@ Ni Al-LDH/distillation water in a certain volume. Microspheres were incubated for 15 min with enzyme and was shaken slowly at room temperature. The supernatant was carefully removed by micropipette by a magnet. Quantification of protein concentration of the isolated supernatant was performed by Bradford assay.

##### 3.2.8.2 Purification of his-tagged proteins from cell lysate by microsphere

The microsphere was incubated in a certain volume with 10–60 μl of His-tagged protein from cell lysate at 4 °C with shaking 100 rpm for 15 min. The pellet was separated by the magnet and washed with a washing buffer (50 mM Tris-HCl pH 7.8). Imidazole solution (0.1 g/ml) was added to the microsphere with shaking 100 rpm for 20 min to release the protein. The resulting supernatant was decanted and PheDH purification was confirmed by the SDS-PAGE method by checking the protein mass and subsequently, purified protein with a specific enzyme assay and a protein quantification method (Bradford reagent) to determine the concentration of captured protein by the microsphere (Fig. S13). It is possible to use this microsphere in removing mutant protein from serum using this microsphere, due to the surface of SiO_2_ and nickel, in conjugation with the molecular template of phenylalanine dehydrogenase in an aqueous solution with an imprinted polymer. The treatment of phenylketonuria with the help of gene therapy is possible with the help of magnetic nanoparticles [[115], [116], [117], [118]].

## 4 Conclusion

Fe_3_O_4_@SiO_2_@ Ni–Al LDH microsphere can be used in protein separation because microspheres provide docking sites for the (His)-tagged proteins. The result of this work revealed that the Fe_3_O_4_@SiO_2_@Ni Al-LDH microsphere has changed the secondary structure of PheDH so that the circular dichroism (CD) demonstrated that the percentage of α helix and β strand were significantly different in the presence of microsphere. Fe_3_O_4_@SiO_2_@ Ni Al-LDH microsphere displays an excellent interaction between the microsphere and PheDH with a binding capacity of 241 μg/mg. The microsphere exhibits good results in terms of influence on kinetic parameters of enzyme and increase of temperature stability of the enzyme. In the present investigation, Fe_3_O_4_@SiO_2_@ Ni–Al LDH microsphere changed the activation energy of the enzyme. Overall, this study indicates that Fe_3_O_4_@SiO_2_@ Ni–Al LDH microsphere is effective on kinetic and structural properties of PheDH and may have potential applications in fields of biosensor design, protein separation, and biomedical science.

## Author contribution statement

Mozhgan Amirahmadi: Conceived and designed the experiments; Performed the experiments; Wrote the paper.

Saman Hosseinkhani: Analyzed and interpreted the data.

Morteza Hosseini, Paricher Yaghmei, Akbar Heydari: Contributed reagents, materials, analysis tools or data.

## Funding

This work was supported by 10.13039/501100008257Research Council of Tarbiat Modares University {IG/39803}.

## Data availability statement

No data was used for the research described in the article.

## Declaration of competing interest

The authors declare that they have no known competing financial interests or personal relationships that could have appeared to influence the work reported in this paper.
