# Supplementary material for: Fe3O4@SiO2@NiAl-LDH microspheres implication in separation, kinetic and structural properties of phenylalanine dehydrogenase
Source: Heliyon. 2023 Sep 3;9(9):e19429. doi: 10.1016/j.heliyon.2023.e19429 (PMC10558515; doi:10.1016/j.heliyon.2023.e19429)
Supplement: Multimedia component 1 [file mmc1.docx]

Fe_3_O_4_@SiO_2_@NiAl -LDH microspheres implication in separation, kinetic and structural properties of phenylalanine dehydrogenase

Mozhgan Amirahmadi^a^, Saman Hosseinkhani*, Morteza Hosseini^b^,Parichehreh yaghmaei^c^, Akbar Heydari ^d^

*^a^ Department of Biochemistry, Faculty of Basic Sciences, Science and Research Branch, Islamic Azad University, Tehran, Iran*

*E-mail: mozhgan.amirahmadi@srbiau.ac.ir*

*^a^** *Department of Biochemistry, Faculty of Biological Sciences, Tarbiat Modares University, Tehran 14115-175, Iran*

**Corresponding author.* Fax: +98-21-88009730; Tel: +98-21-82884407;

*E-mail:* [*saman_h@modares.ac.ir*](mailto:saman_h@modares.ac.ir)

*^b^ Department of Life Science Engineering, Faculty of New Sciences & Technologies, University of Tehran, Tehran 1417614418, Iran*

*smhosseini@khayam.ut.ac.ir*

*^c^Department of Biology, Faculty of Basic Sciences, Science and Research Branch, Islamic Azad University, Tehran, Iran*

[*yaghmaei.pa@gmail.com*](mailto:yaghmaei.pa@gmail.com)

*^d^ Chemistry Department, Tarbiat Modares University, P.O. Box 14155-4838, Tehran, Iran*

[*heydar_a@modares.ac.ir*](mailto:heydar_a@modares.ac.ir)

| **Table of contents** |
| --- |
| **Subject Page** |
| **Figure S1.** Images of preparation of preculture from BL21 bacteria.**..................................................................**S3 **Figure S2.** Images Figure S2. Stock preparation of BL21 bacteria............................................................................S4  **Figure S3.** Preparation of culture medium for bacterial growth...............................................................................…S5  **Figure S4**. Expression of phenylalanine dehydrogenase protein from bacteria............................................................S6  **Figure S5**. Purification steps of phenyl dehydrogenase protein from bacteria.............................................................S7  **Table S1**. The zeta potential for Fe_3_O_4_@SiO_2_@ Ni Al- LDH microsphere..................................................S8  **Table S2**. average size for Fe_3_O_4_@ SiO_2_@ Ni Al LDH microsphere............................................................S8  **Figure S7**. Line weaver-Burk plot shows Km and Vm for PheDH enzyme............................................... S9  **Figure S8**. Line weaver-Burk plot demonstrates km and Vm for PheDH in presence of 0.006 µM concentration of Fe_3_O_4_@ SiO_2_@ Ni Al LDH microsphere. ..................................................................... S10  **Figure S9**. Line weaver-Burk plot shows km and Vm for enzyme in 0.013 µM concentration 0f  Fe_3_O_4_@ SiO_2_@ Ni Al LDH microsphere. ................................................................................................ S10  **Figure S10**. Figure S9. Lineweaver-Burk plot drown for Phe DH enzyme in presence of 0.026 µM concentration of Fe_3_O_4_@ SiO2@ Ni Al- LDH microsphere........................................................................... S11  **Figure S11**. 11a.optimum temperature curve of enzyme and 11b.Optimum temperature curve of the enzyme in the presence of 0.026Μm microsphere concentration.............................................................S12. |
| **Figure S12.** Thermal stability curve of enzyme (10A). thermal stability curve of the enzyme in the presence of 0.026mM microsphere concentration(10B). .......................................................................................S13  Figure S13. SDS-PAGE to confirm PheDH separation using Diaphorase and Luciferase as molecular marker or ladder……………………………………………………………………………………S14 |
|  |

preculture

LB medium/10ml

Incubate/37^◦^ c

12-16h

over night

Kanamycin

(50mg/ml) /10 µl

add stock /10 µl

**Figure S1.** Preparation of preculture from BL21 bacteria

Homogenize/pipette

Centrifuge/1000rpm/1min

stock

preculture

Vials(1ml)

Freeze (- 80) ^◦C^

^0^c

Adding /glycerol/ 60% /10 µl

Remove/500 µl

**Figure S2.** Stock preparation of BL21 bacteria

culture

2xYT medium

250 ml

Kanamycin 250 µl

3-4h

Preculture

1000 µl

Incubator/37 ^◦^C

Shake/180rpm

**Figure S**3. culture preparation of BL21 bacteria

Expression

2xYT medium

250 ml

Shake/180 rpm

18^◦^c

Lactose

5ml/8Mm/v_t_250ml

Incubation/overnight

**Figure4.** Expression of phenylalanine dehydrogenase protein from bacteria

purification

sediment

supernatant

Centrifuge/14000g/4^◦^c

Transfer/falcon

15min

pellet

supernatant

Centrifuge

6000g/4^◦^c

remove

supernatant

supernatant

Pellet/ in/ ice

pellet

sonication

pipetage

Centrifuge

Lysis buffer/1ml

13tims/interval/40s

20min/14000g

**Fiure5.** Purification steps of phenylalanine dehydrogenase protein from bacteria

**Table S1**.The zeta potential for Fe_3_O_4_@SiO_2_@ Ni Al- LDH microsphere


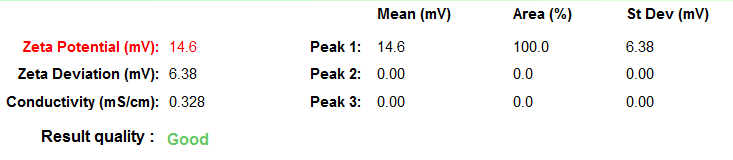


**Table S2**. average size for Fe_3_O_4_@ SiO_2_@ Ni Al LDH microsphere


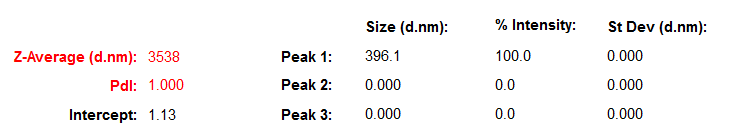


Figure S7. Line weaver-Burk plot shows Km and Vm for PheDH enzyme

1/vm

Figure S8. Line weaver-Burk plot demonstrates km and Vm for PheDH in presence of 0.006 µM concentration of Fe_3_O_4_@ SiO_2_@ Ni Al- LDH microsphere.

1/vm1

Figure S9. Lineweaver-Burk plot shows km and Vm for enzyme in 0.013 µM concentration 0f

Fe_3_O_4_@ SiO_2_@ Ni Al- LDH microsphere.

-1/Km

1/Vm

Figure S10.lineweaver-Burk plot drown for Phe DH enzyme in presence of 0.026 µM concentration of Fe_3_O_4_@ SiO_2_@ Ni Al- LDH microsphere

**(A)**

**(B)**

Figure S11. optimum temperature curve of enzyme and (A). Optimum temperature curve of the enzyme in the presence of 0.026 mM microsphere concentration(B).

A

B

Figure S12. Thermal stability curve of enzyme (A). thermal stability curve of the enzyme in the presence of 0.026mM microsphere concentration(B).


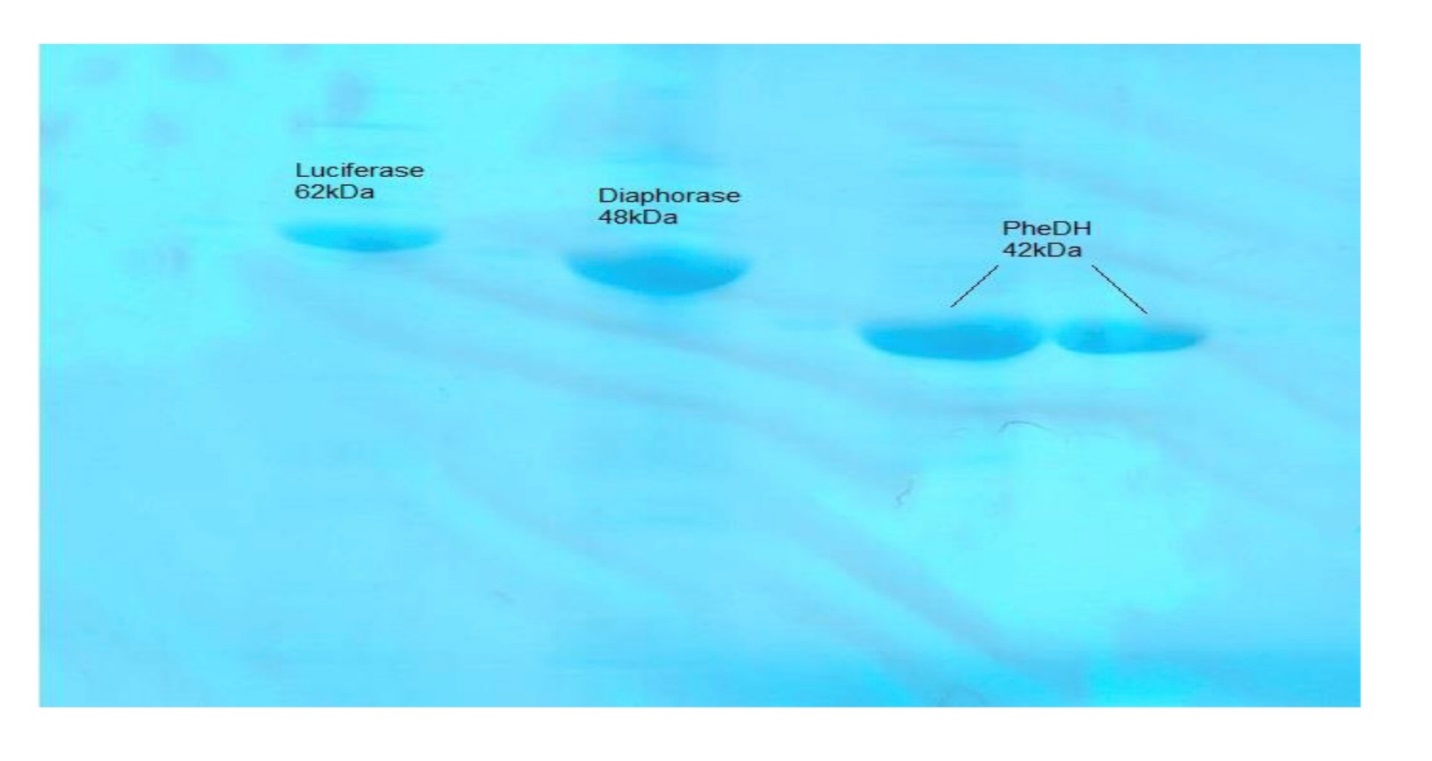


Figure S13. SDS-PAGE to confirm PheDH separation using Diaphorase and Luciferase as molecular markers for comparison.
